# An Investigation of Human Errors in Medication Adverse Event Improvement Priority Using a Hybrid Approach

**DOI:** 10.3390/healthcare9040442

**Published:** 2021-04-09

**Authors:** Min-Chih Hsieh, Po-Yi Chiang, Yu-Chi Lee, Eric Min-Yang Wang, Wen-Chuan Kung, Ya-Tzu Hu, Ming-Shi Huang, Huei-Chi Hsieh

**Affiliations:** 1 Department of Industrial Engineering, University of Shanghai for Science and Technology, Shanghai 200093, China; g9674019@cycu.org.tw; 2 Department of Industrial Engineering and Engineering Management, National Tsing Hua University, Hsinchu 30013, Taiwan; s105034571@m105.nthu.edu.tw (P.-Y.C.); mywang@ie.nthu.edu.tw (E.M.-Y.W.); 3 School of Design, South China University of Technology, Guangzhou 510006, China; 4 Nursing Department, Hsinchu Mackay Memorial Hospital, Hsinchu 30071, Taiwan; 6444@mmh.org.tw (W.-C.K.); m240@mmh.org.tw (M.-S.H.); 6405@mmh.org.tw (H.-C.H.); 5 Pharmacy Department, Hsinchu Mackay Memorial Hospital, Hsinchu 30071, Taiwan; l135@mmh.org.tw

**Keywords:** human error, medication adverse events, Human Factor Analysis and Classification System (HFACS), Analytical Hierarchy Process (AHP), Technique for Order of Preference by Similarity to Ideal Solution (TOPSIS)

## Abstract

The aim of this study was to analyze and provide an in-depth improvement priority for medication adverse events. Thus, the Human Factor Analysis and Classification System with subfactors was used in this study to analyze the adverse events. Subsequently, the improvement priority for the subfactors was determined using the hybrid approach in terms of the Analytical Hierarchy Process and the fuzzy Technique for Order of Preference by Similarity to Ideal Solution. In Of the 157 medical adverse events selected from the Taiwan Patient-safety Reporting system, 25 cases were identified as medication adverse events. The Human Factor Analysis and Classification System and root cause analysis were used to analyze the error factors and subfactors that existed in the medication adverse events. Following the analysis, the Analytical Hierarchy Process and the fuzzy Technique for Order of Preference by Similarity to Ideal Solution were used to determine the improvement priority for subfactors. The results showed that the decision errors, crew resource management, inadequate supervision, and organizational climate contained more types of subfactors than other error factors in each category. In the current study, 16 improvement priorities were identified. According to the results, the improvement priorities can assist medical staff, researchers, and decisionmakers in improving medication process deficiencies efficiently.

## 1. Introduction

### 1.1. Medication Adverse Events

Medication errors pose serious threats to health care systems and patients around the world [1,2,3,4]. These errors can occur in the process of medication, posing considerable health and financial burdens to patients, hospitals, and healthcare systems [4,5]. Previous investigations into these issues have indicated that medication errors account for 18% of medical errors in the state of Pennsylvania in the United States and 30.8% of medical errors in Taiwan [6]. Further, according to the Taiwan Patient-safety Reporting system (TPR), medication errors accounted for 33.5% of medical errors in the first three seasons of 2020 [7,8,9]. Elliott et al. [10] indicated that avoidable medication adverse events cost approximately GBP 98,462,582 per year and cause up to 1708 deaths in England. The World Health Organization announced in 2017 that the level of severe and avoidable harm related to medication issues should be reduced by 50% in the next 5 years [11]. Medication errors are thus a global issue and preventing them could increase patient safety and help avoid unnecessary expenses.

Medication errors can be defined as “*a failure in the treatment process that leads to, or has the potential to lead to, harm to the patient*” [12,13]. In order to prevent medication adverse events, analyzing and classifying them is an important step that can help researchers to understand how medication errors occur. Previous studies have analyzed medication adverse events and have classified these error factors from the perspective of defective behaviors or psychological mechanism theories [5,8,9,10,11,13,14,15]. Kaushal et al. [14] reviewed 10,778 medication orders and 616 medication errors were found. The results showed that most of the medication errors occurred during the drug ordering process and were related to defective behaviors, such as incorrect dosing and the failure to use anti-infective drugs and intravenous medications. Similarly, Pham et al. [5] classified the error factors from the aspect of defective behaviors. They analyzed 496 reports from the Emergency Department (ED) and found 13,932 medication errors, with error types including improper dose or quantity, not following procedure or protocol, and poor communication. On the other hand, the results of Pham’s study also classified the error factors from the aspect of psychological mechanism. The authors found that distractions and increasing workload might lead to the occurrence of medication adverse events. Freund et al. [15] analyzed 34 ED adverse events using five psychological mechanism error types, such as violation, procedural, communication, proficiency, and decision.

In fact, adverse events frequently occur accompanied by a series of unsafe conditions [16], and the most important part of error analysis is to interpret why and how the errors occur. However, various studies related to medication error analysis have only analyzed medication adverse events from the psychological and behavioral point of view, but less consideration has been given to the latent errors (such as management problems, organizational issues, etc.) that might lead to accidents indirectly [6]. Therefore, analyzing adverse medication events should not only focus on the phenomenon of events (obvious personal defective behaviors or psychological mechanisms) but also analyze the event to find the causal factors.

### 1.2. Human Factors Analysis and Classification System

From the error analysis perspective, identifying the causal factors behind an accident is important. There are many classification models can be used to analyze human errors that hide in the health care system, and the Human Factors Analysis and Classification System (HFACS), proposed by Wiegmann and Shappell [17], is a well-known error classification framework that is widely used in many fields such as aviation safety, patient safety, nuclear power plants, railway safety, and the chemical industry [4,6,18,19,20,21,22,23]. For instance, Kilic and Gümuüs [22] used the HFACS framework to analyze 30 commercial airplane crashes that occurred over the past 5 years. The results showed that skill-based errors, physical environment, crew resource management, and resource management were highest frequency factors, from level 1 to level 4 of HFACS, respectively. Wang et al. [23] applied the HFACS framework to analyze 101 accidents in the chemical industry. Cohen et al. [19] analyzed the surgical near-miss events by HFACS, and the results showed that skill-based errors, tools/technology, inadequate supervision, and resource management were the most important error factors in the surgical near-miss events. The HFACS has provided a framework that follows the Swiss cheese model for accident analysis, which corresponds to four error category levels: Level 1 to level 4 represent the categories of unsafe acts, preconditions for unsafe acts, unsafe supervision, and organizational influences, respectively. Different types of error factors are included in each category. For example, decision errors is one of the error factors in the unsafe acts category, and adverse mental state is one of the error factors in the preconditions for unsafe acts. Inadequate supervision is one of the error factors in unsafe supervision, and organizational process is one of the error factors in organizational influences. Using decision errors as an example, selected incorrect treatment procedure and misinterpretation of patient information may result in decision errors. Another is adverse mental state, which related to the psychological mechanism of medical staff. Task overload, distraction, and stress may lead to the occurrence of medication errors. Thus, the HFACS framework not only includes the defective behaviors and psychological mechanism error factors but also involve the management and organizational issues in the system.

According to the discussed above, two problems related to human error analysis were observed. The first problem is that, although previous studies have applied the HFACS to analyze the causal factors in accidents successfully, the error factors classified as adverse behaviors or situations in these studies may not give an in-depth explanation of the accidents. In fact, various subfactors were classified under each error factors by Wiegmann and Shappell [17], which could specify the inappropriate behavior displayed in the accident rather than providing the concept of unsafe human behavior. For instance, timing error is one of the subfactors related to skill-based errors. It can show unsafe behaviors in a specific way [6,24]. Overall, previous studies have devoted considerable effort toward using the HFACS error factors instead of the subfactors to analyze accidents, and this approach has probably prevented us from acquiring some important information from the accidents. Thus, using the subfactors of the HFACS to analyze medication adverse events is one purpose of this study, which might assist researchers and medical staff to better understand how and why errors occur. The second problem is that previous studies have used the occurrence frequency (or serious level) to record the error factors [6,15,19,25,26]. However, using “frequency” as an indicator to represent the importance of error factors or improvement priority of error factors may not be an appropriate and efficient way to prevent the occurrence of human errors [18]. Different conditions should be considered to determine the error factor improvement priority, such as the Severity Assessment Code Matrix, which uses the occurrence frequency and serious level simultaneously to determine the level of patient injury.

### 1.3. Technique for Order Preference by Similarity to Ideal Solution

Since the error factor improvement priority in this study is not only analyzed by one-dimension (frequency of error factors) but by multiple dimensions, this study can be regarded as a Multiple-Criteria Decision-Making (MCDM) problem. To conveniently deal with the MCDM problem, Hwang and Yoon [27] proposed a method called the Technique for Order Preference by Similarity to Ideal Solution (TOPSIS), which has been widely applied in the fields of aviation, health care, marketing, and the supply chain [18,28,29,30,31,32,33]. The idea of TOPSIS is that the geometric distance between the selected alternative and the positive idea solution should be the shortest, while the geometric distance should be longest between the selected alternative and the negative idea solution [33]. However, the vagueness or ambiguity problems that exist in decision-making process are hard to deal with using TOPSIS. Hence, previous studies have integrated TOPSIS and fuzzy set theory as fuzzy TOPSIS to evaluate multicriteria decision-making problems, which can assist the researchers in bringing the information that cannot be quantified, as well as incomplete, unavailable, and partially uncertain factors, into the decision model [18,30,33]. In addition, determining the weight of criteria is one of the important steps in TOPSIS which can affect the ranking of the alternatives. However, the TOPSIS method does not provide a calculation method for how to obtain the weight of each criterion. In order to solve this problem effectively, the Analytical Hierarchy Process (AHP) [34] can be used to determine the criteria for TOPSIS. The AHP is a simple method for efficiently assessing both qualitative and quantitative measurements [35,36]. It can be integrated with other methods (i.e., TOPSIS) to solve complicated decision-making problems. Dağdeviren et al. [37] applied AHP to determine the weight of the criteria for TOPSIS; Kumar et al. [38] integrated fuzzy set theory, AHP, and TOPSIS to investigate the usable security of web applications; Jain et al. [39] used fuzzy AHP, and TOPSIS to investigate supplier selection in the automotive industry; and Yucesan and Gul [40] evaluated hospital service quality by the hybrid method in terms of fuzzy AHP and fuzzy TOPSIS. It can be seen that these three methods have been integrated successfully and applied to solve problems among various fields.

### 1.4. Purposes of This Study

Since previous studies have not analyzed the medical/medication adverse events in a more specific and in-depth way, and have not applied specific conditions to determine the improvement priority for enhancing patient safety as discussed above, a subsequent study is necessary. Therefore, the purpose of this study was to analyze adverse medication events in Taiwan using the HFACS framework with subfactors and evaluate the improvement priority for subfactors by applying AHP and fuzzy TOPSIS. In order to achieve the purposes of this study, we followed four main steps: (1) Select the appropriate cases related to medication errors from the TPR system, (2) analyze medication errors using HFACS, (3) determine the weight for each criterion using AHP, and (4) evaluate the improvement priority of error factors based on the criteria using fuzzy TOPSIS. The detail process of these steps is presented in the next sections, respectively. By integrating HFACS, AHP, and fuzzy TOPSIS, the results of this study only identify the error factors but also uncover the subfactors among the adverse medication events and identify the improvement priority for subfactors based on different criteria rather than using a single criterion to determine the importance of the error factors and subfactors. The results are expected to help researchers and medical staff to better understand why human errors occurred and how to reduce them efficiently while improving patient safety.

## 2. Materials and Methods

### 2.1. Adverse Medication Events

This was a retrospective study conducted at a regional hospital with over 600 hospital beds in Hsinchu, Taiwan, focused on finding the medication error factors and improvement priority for the subfactors. Adverse medical events related to medication errors were selected from the TPR system to conduct this research. This study was approved by the Institutional Review Board (approved number: 16MMHIS171e) of the cooperation hospital.

The study included all adverse medical events from the TPR system as the considered cases. Thus, downsizing the number of adverse events and ensuring that the adverse events selected were related to medication errors were needed. The study adopted 2 steps to effectively identify adverse medical events associated with medication errors. A vice director of the nursing department with over 20 years of experience working in the hospital and an expert from the field of human factors and ergonomics were recruited to identify the initial adverse medical events. The inclusion criteria of the events were as follows. First, the adverse medical events category had to involve a medication error. Since the operations, nursing processes, and medications used by different hospitals vary, not all of the events that met the first criteria were likely to occur in the cooperating hospital. Second, the selected adverse medication events from the first scenario needed to have a substantial opportunity to happen in the cooperative hospital. These steps ensured that the selected adverse medical events associated with medication errors were used in this study.

### 2.2. Medication Error Analysis Process

The final pool of adverse medication events was produced following a step-by-step selection process. The experts were recruited from the cooperating hospital in this study, and the chosen adverse medication events were analyzed with the help of 3 nurses (including 1 vice director from the nursing department), 3 pharmacists (including 1 technical director from the pharmacy department), and 2 human factors and ergonomics experts. All of them had more than 15 years of work experience at the cooperating hospital, and the human factors and ergonomics experts had more than 2 years of work experience related to human error analysis at National Tsing Hua University.

**Figure 1:**
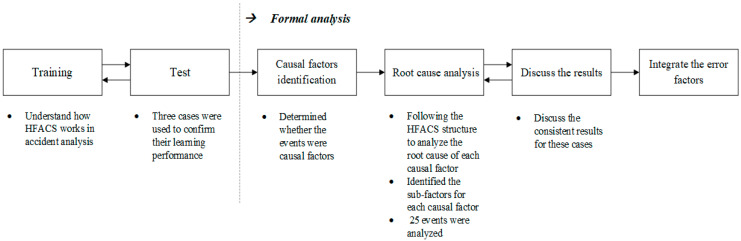
Process of error factor and subfactor analysis (Note: HFACS in this figure represent the Human Factors Analysis and Classification System).

The analysis process is shown in Figure 1. Before executing the formal analysis, we spent 10 hours helping the 6 experts (3 nurses and 3 pharmacists) understand how the HFACS works in accident analysis and confirm their learning performance. At the beginning of the formal analysis, the experts were required to determine the causal factors for the 25 adverse events (not including the 3 training cases). The experts were then asked to further analyze the root cause of each causal factor following the HFACS structure. The analysis process of root cause was followed as per Hsieh et al. [33]. The causal factors were classified by the experts into level 1 of the HFACS, and then the subfactors were identified for each causal factor subsequently. The error factors and subfactors in HFACS level 2 were analyzed based on the causal factors in level 1. The other error factors and subfactors in levels 3 and 4 were analyzed in the same manner. The types of error factor and subfactor were recorded.

### 2.3. Criteria Identification

The criteria are the terms that were used to evaluate the performance of the error factor under the given conditions in this study. When setting the criteria for the improvement priority of error factors, it is necessary to consider whether these criteria can reflect the urgency and economy of improving error factors to help researchers, medical staff, and hospital decision-makers to start improving the error factors [33]. Since improvement priority was one of the main outcomes expected from this study, the criteria used in this study were determined by the members of the expert team based on their duties and past experiences. The following 3 criteria related to the improvement priority were established.

Influence: The severity of the errors in the medication adverse events.
Time: The human error reduction time.
Cost: The cost of reducing human errors.

From the perspective of patient safety, the influence of adverse events and how fast the adverse events can be improved are critical issues affecting the patient safety of hospitals and health care systems. The influence of adverse events represents the severity of the errors, which may threaten patients’ life or safety. The reduction time of human errors represents the improved efficiency: The shorter the reduction time, the higher the improved efficiency, and the better it can prevent adverse events from happening again. Moreover, from the perspective of the decision-maker, they must not only reduce the occurrence of adverse events but also consider the cost of improving these adverse events. Thus, according to the opinions and experience of the experts, “Influence,” “Time,” and “Cost” were used as the criteria in this study to evaluate improvement priority of error factors.

### 2.4. AHP Method

**Figure 2:**
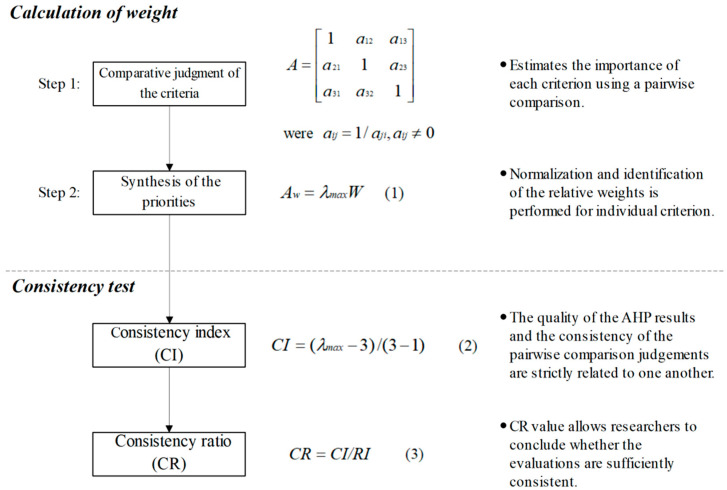
Execution steps of the Analytical Hierarchy Process (AHP) method (Note: The code (1), (2), (3) in this figure represent the formula number used in the AHP calculation process; RI in this figure represent Random Consistency Index).

AHP was applied in this study to determine the weights of the criteria used in the TOPSIS calculation process, which contained 2 main steps. The first step was to estimate the importance of each criterion through a pairwise comparison by the members of the expert team, and a standardized comparison scale with 9 levels (1 represents equally important, and 9 represents extremely more important) was applied in this pairwise comparison process. Let $C=\{C_{j}|j=1,\;2,\;3\}$ be the set of criteria. The pairwise comparison on 3 criteria can be presented in a (3×3) evaluation matrix, A. Every element $a_{ij}$ (i=1, 2, 3; j=1, 2, 3) of the matrix A is the quotient of weights of the criteria, which is shown in Figure 2. In the second step, normalization and identification were implemented for the relative weights of each criterion. The relative weights are given by the right eigenvector (*W*) corresponding to the largest eigenvalue ($\lambda_{max}$), which is shown in Figure 2. After calculating the weight of each criterion, the consistency test was performed to evaluate the quality of AHP results. If the final consistency ratio (CR) exceeded the acceptable upper limit of 0.1, the assessment process was repeated to ensure consistency. Consistency measurement can be used to evaluate the consistency of decision-makers and the overall hierarchy [37].

### 2.5. Fuzzy Theory and TOPSIS

The linguistic values were used by fuzzy set theory to represent the decision-maker’s selection, which then was converted into a fuzzy number to solve the MCDM problems. The current study applied triangular fuzzy numbers (TFN) as an effective approach in the adverse medication events analysis process to formulate decision issues related to subjective and imprecise information [41,42]. With regard to the linguistic variables, Chen and Hwang [43] developed a set of 8 scales based on TFN, each containing different numbers of linguistic variables with an exact value. For instance, the scale number 6 involve 7 linguistic variables, and the scale number 8 involve 11 linguistic variables. The current study employed 5 linguistic variables (scale number 4) to assess the levels in each TOPSIS criterion. The expert team in this study applied the linguistic variables with a 5-level scale to evaluate each subfactor based on the 3 criteria. After the subfactor evaluation process, defuzzification was carried out to translate the fuzzy numbers to the exact values. Chen and Hwang [43] provided the results of defuzzification for the 5-level scale in their research. The results showed that the five exact values of the five linguistic values are 0.091, 0.283, 0.5, 0.717, 0.909 (0.091 represents very low, and 0.909 represents very high), respectively. Thus, this study used the results directly for the subsequent evaluation of improvement priority.

**Figure 3:**
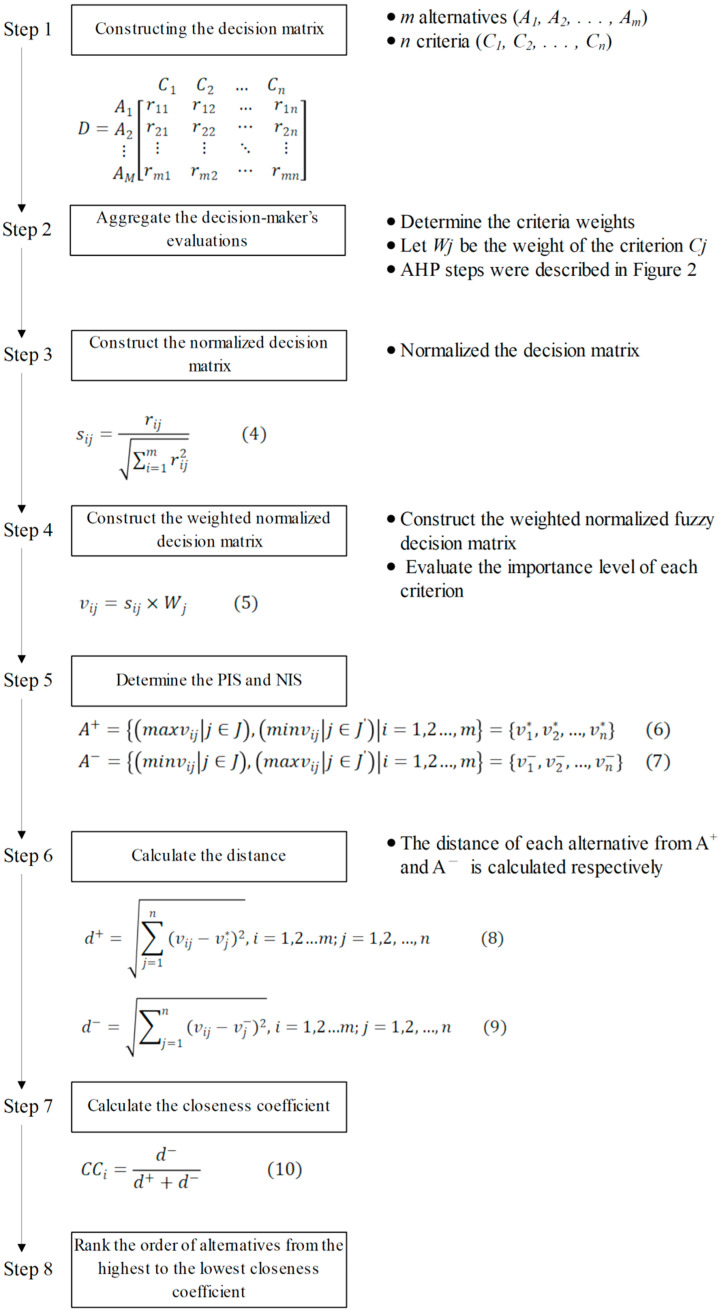
Application process of Technique for Order of Preference by Similarity to Ideal Solution (TOPSIS) (Note: The codes (4) to (10) in this figure represent the formula number used in AHP calculation process; PIS and NIS in this figure represent Positive Ideal Solution and Negative Ideal Solution, respectively).

With TOPSIS, the main objective is to generate a ranking of potential alternatives [30]. The potential alternatives in this study represent the sub-actors of the HFACS which were found from medication adverse events by the experts. The 6 experts evaluated these potential alternatives based on the 3 criteria (influence, time, and cost) using the 7 linguistic variables. The assessment results of the potential alternatives can be denoted as the decision matrix of TOPSIS. Herein, the next step is to identify the Positive Ideal Solution (PIS) and the Negative Ideal Solution (NIS), and then measure the distance between each potential alternative and the ideal solution. The alternative with the smallest distance to the PIS and the largest distance to the NIS was selected as the solution to the TOPSIS problem. The TOPSIS steps used in this study are based on the previous studies by Kannan et al. [30] and Hsieh et al. [33] and the application process is shown in Figure 3.

## 3. Results

### 3.1. Medication Adverse Event Analysis

In the current study, 157 medical adverse events related to medication errors were selected from the TPR system and analyzed. Of these, 46 events related to medication errors were identified at the first selection step. From the 46 medication adverse events, 25 were identified as the events at the second selection step. All of these 25 events were likely to occur in the cooperating hospital. After error analysis, the types of subfactors identified by the six experts were recorded separately. Table 1 lists the 76 types of subfactors in each HFACS error factor. According to the results, the decision errors (8 types), crew resource management (10 types), inadequate supervision (3 types), and organizational climate (8 types) contained more types of subfactors than other error factors in each category, respectively.

### 3.2. AHP Analysis Results

The criteria weights used in TOPSIS were calculated using the AHP method. The values in Table 2 represent the original data extracted from the individual pairwise comparison matrix to calculate the group pairwise comparison matrix by geometric means and then standardize the pairwise comparison matrix. After calculating the average number of each row vector, the results of the weights for each criterion were obtained, as shown in Table 3. The CR value of the pairwise comparison matrix was calculated by Formula (3) as 0.005 < 0.1. Thus, the weights of three criteria were consistent and could be applied to the TOPSIS process.

### 3.3. Results of Fuzzy TOPSIS

The subfactors of the skill-based errors were used as an example to illustrate how fuzzy TOPSIS worked in this study. Table 4 shows the fuzzy decision matrices of the skill-based errors, which represent the average value of the exact value from the judgement of each expert. Following the calculation and application process shown in Figure 3, the weighted normalized decision matrix, distance of each criterion to PIS and NIS, closeness coefficient, and ranks obtained by each criterion were calculated (Table 5 and Table 6). The fuzzy decision matrix (Table 4) was normalized by Formula (4) (step 3 in Figure 3), and the weights of the criteria were multiplied with the normalized matrix to form a weighted normalized fuzzy decision matrix using Formula (5) (step 4 in Figure 3). Then, the PIS and NIS were determined by following Formula (6) and Formula (7) (step 5 in Figure 3), respectively. Table 5 shows the weighted and normalized decision matrices with the PIS and NIS. Next, the distance of the criteria from the PIS and NIS was calculated using the Formula (8) and Formula (9) (step 6 in Figure 3). In order to rank the improvement priority of subfactors based on their closeness to the PIS and remoteness to the NIS, the closeness coefficient (CC) was calculated using Formula (10). Table 6 shows the distance of the criteria from the PIS and NIS, as well as ranks of subfactors for “skill-based errors.” The ranks of the top three subfactors for each error factor after executing the fuzzy TOPSIS calculation are listed in Table 7.

## 4. Discussion

Subfactor priority based on three criteria was provided by this study, which was the main finding of this study. Analyzing an improvement strategy is a decision-making problem. Additional decision criteria can potentially make the results more objective. Fuzzy TOPSIS allowed the use of three criteria in terms of influence, time, and cost to determine the improvement priority of each subfactor, while most previous studies [3,6,24,44,45] have used only the occurrence frequency as a criterion. Although these studies have identified the error factors that directly or indirectly resulted in adverse medical events, the calculation of frequency of error factors may not present an efficient way to reduce errors.

This is not the first study to use the MCDM method to increase patient safety. Wang and Chou [31] selected the human factor problems from previous literature and applied fuzzy TOPSIS to assess it in patient safety management. Contrary to Wang and Chou’s work, the current research is a retroactive study, representing the subfactors analyzed from real adverse medication events. Therefore, the improvement subfactor priority in the current study is more specific to hospitals than that in Wang and Chou’s study. In addition, Hsieh et al. [33] used fuzzy TOPSIS and the AHP method to determine the important human error factors in emergency departments. As in the current study, Hsieh et al. [33] used fuzzy TOPSIS with three criteria in their study. However, the purpose of these two studies is different. The criteria used in these two studies were also different. Hsieh’s study focused on finding the important error factors, so the criteria used in their study were related to the influence of the error factors, such as reproducibility and preventability of the error factors. Hence, the results of Hsieh’s study differ from those of the current study.

Previous studies [6,18,33] have applied the HFACS to identify the error factors in aviation maintenance tasks, medical adverse events in emergency departments, and medication adverse events. However, the current study applied the HFACS to analyze the medication adverse events and also used the HFACS subfactors to identify human errors. Therefore, the results in the current study are more specific than those in the three other studies. In addition, Li et al. [20] used HFACS combined with the Systems–Theoretical Accident Modelling and Processes and human information process to analyze the error factors in railway accidents. Havle and Kılıç [46] applied HFACS combined with fuzzy AHP to analyze gross navigation errors. The current study also combined HFACS with AHP and fuzzy TOPSIS to determine the improvement priority for error factors. Hence, HFACS provides a framework that can combine with other methods to analyze accidents based on the demands and the purposes of the study.

Actually, more criteria should be considered to determine the improvement priority of error factors rather than using one criterion. In the results of the current study, a skill-based error factor was used as an example. An efficient way to reduce skill-based errors is to decrease the defects in the conducted sequence item out of order first, and then reduce the negative effects of the work or motion at improper speed. Using the nursing task as an example, although the nurse-to-patient staffing ratio in Taiwan met the standard requirement (the 2016 Hospital Accreditation Standards that announced by Joint Commission of Taiwan), the ratio was still higher than that in other countries such as the United States and Japan [47,48]. This reflects the fact that the medical staff are overloaded and busy when they are on duty, forcing them to complete their routine work at a fast pace. However, from the perspective of improvement priority, addressing the understaffing defect is not the most efficient way to reduce skill-based errors in adverse medication events because that is a nationwide medical system problem.

## 5. Conclusions

This study applied the AHP, fuzzy TOPSIS, and HFACS to analyze the error factors behind adverse medication events. The improvement priority of subfactors was also identified. The results showed that 76 types of subfactors were analyzed from adverse medication events, and 16 improvement priorities calculated by AHP and fuzzy TOPSIS were summarized in the study.

There are three limitations to this study. First, since the medication process, nursing tasks, and organization are different in different hospitals, the results of this study might not be generalizable to all Taiwan hospitals. Second, as this was a retrospective study, it was extremely difficult to know the exact behaviors of the medical staff and the state of medical environments at the time the error occurred. The study depended on the experience of six experts to analyze and interpret the adverse medication events. For these reasons, the results of this study might not completely reflect the real medical situations. Finally, although the experts had at least 10 years of work experience in the hospital, they were still all first-line medical staff and not actually involved in the decision-making and operation of the hospital. Therefore, while evaluating the weights of the three criteria, the experts tended to pay more attention to the influence criterion rather than the other two criteria, which caused the weights of the influence criterion to be relatively higher than the cost and time criteria and further affected the results of this study. If comments from the hospital managers could be added to this study, the results may differ.

Increasing patient safety is an important issue worldwide [49,50], and reducing human errors in the medication process was the goal of this study. However, enhancing medication and patient safety have never been the responsibilities of the medical staff or hospitals alone. Unsafe behavior or lack of knowledge about medications by patients is also a potential threat to patient safety. Therefore, the important issues to consider in future research include analyzing the medical adverse events related to unsafe behavior by patients or their lack of knowledge about medications, classifying the error factors by patients, and integrating these findings with the HFACS. By doing so, the medical staff and researchers may be able to understand the causes of adverse medication events more comprehensively and prevent the occurrence of adverse medication events.

## Figures and Tables

**Table 1:** Subfactor types that exist in adverse medication events.

Human Factor Analysis and Classification System (HFACS) Framework				
Unsafe Acts				
Decision errors		Skill-based errors	Perception errors	Violations
Inadequate risk assessment	Selected incorrect procedure	Safety checklist error	Misperceived patient factors (e.g., strength/weight-bearing)	Violation of policy/procedures/standard of care
Critical-thinking failure	Failure to prioritize task	Work or motion at improper speed	Misinterpreted/misread equipment	Distracting behavior
Caution/warning ignored or misinterpreted	Improper use of instrument, equipment, personal protective equipment (PPE), and/or materials	Lapse of memory/recall for all or part of a procedure		Taking shortcuts (not otherwise specified)
Inadequate report provided		Conducted sequence item out of order		Failure to follow orders
Misinterpretation of information		Poor technique (e.g., intubation, central line insertion)		
**Preconditions for Unsafe Acts**				
Technological environment	Adverse mental states	Adverse psychological states	Physical/Mental limitations	Crew resource management
Inadequate/defective warnings/alarms	Task overload	Inadequate rest/sleep	Limited experience/proficiency Information overload	Inadequate communication between providers
Inadequate/Unclear/outdated policies/procedures/checklists	Perceived haste/pressure to complete task	Medical illness	Lack of technical procedural knowledge	Inadequate communication during handoff
Failures of information technology (software and hardware issues)	Inattention/Distraction	Self-medicating	Insufficient reaction time	No or ineffective communication methods
	Complacency/Overconfidence		Lack of aptitude to operate task	Inadequate communication: Staff & patient/family
	Stress (job-related)			Failure to warn/disclose critical information
	Mental fatigue			Failed to use all available resources
				Lack of teamwork
				Verification techniques not used
				Inaccurate information provided
				Confusing/conflicting orders
**Unsafe Supervision**				
Inadequate supervision	Planned inappropriate operations	Failed to correct a problem		Supervisory violations
Inadequate mentoring/coaching/instruction	Failure to match staff competency with the task	Failed to initiate corrective action	Failed to review and revise a policy/procedure	Failed to enforce policies/procedures
Inadequate oversight	Poor crew pairing	Failed to ensure problem was corrected		Authorized hazardous operation
Inadequate training				
**Organizational Influence**				
Resource management	Organizational climate		Organizational process	
Inadequate staffing	Communication	Norms and rules	Operational tempo	Established safety programs/risk management programs
Budgetary constraints	Accessibility of supervisor	Organizational customs	Incentives/punishment	Management’s monitoring and checking of resources, climate, and processes to ensure a safe work environment
Poor equipment design	Visibility of supervisor	Organizational values, beliefs, attitudes	Time pressure	
Failure to correct known design flaws	Hiring, firing, retention		Schedules	
Resources management	Accident investigations		Performance standards	

**Table 2:** Group pairwise comparison matrix for criteria.

Criteria	Influence	Time	Cost
Influence	1.00	2.77	4.90
Time	0.36	1.00	1.40
Cost	0.20	0.71	1.00

**Table 3:** Results of the weights.

Criteria	Weights	$\lambda_{max}$, Consistency Index (CI), Random Consistency Index (RI)	Consistency Ratio (CR)
Influence	0.643	$\lambda_{max}$ = 3.01	0.005 < 0.1
Time	0.215	CI = 0.003	
Cost	0.142	RI = 0.58	

**Table 4:** Fuzzy decision matrices of skill-based errors.

Skill-Based Errors	Influence	Time	Cost
Safety checklist error	0.568	0.477	0.295
Work or motion at improper speed	0.682	0.546	0.477
Lapse of memory/recall for all or part of a procedure	0.591	0.523	0.386
Conducted sequence item out of order	0.773	0.637	0.500
Poor technique	0.591	0.568	0.523

**Table 5:** Weighted and normalized decision matrices with the PIS and NIS of “skill-based errors”.

Skill-Based Errors	Influence	Time	Cost
Safety checklist error	0.253	0.083	0.042
Work or motion at improper speed	0.304	0.095	0.068
Lapse of memory/recall for all or part of a procedure	0.263	0.091	0.055
Conducted sequence item out of order	0.344	0.111	0.072
Poor technique	0.263	0.099	0.075
Positive ideal solution (PIS)	0.344	0.083	0.042
Negative ideal solution (NIS)	0.253	0.111	0.075

**Table 6:** Ranks of subfactors for “skill-based errors.”

Skill-Based Errors	Distance of Each Alternative from Positive Solutions (D^+^)	Distance of Each Alternative from Negative Solutions (D^−^)	Closeness Coefficient (CC*_i_*)	Rank
Safety checklist error	0.091	0.043	0.3211	3
Work or motion at improper speed	0.050	0.053	0.5187	2
Lapse of memory/recall for all or part of a procedure	0.082	0.030	0.2659	4
Conducted sequence item out of order	0.041	0.091	0.6916	1
Poor technique	0.089	0.016	0.1506	5

**Table 7:** Ranks of top three subfactors.

Unsafe Acts			
**Decision errors**	Inadequate risk assessment	Critical-thinking failure	Misinterpretation of information
**Skill-based errors**	Conducted sequence item out of order	Work or motion at improper speed	Safety checklist error
**Perception errors**	Misperceived patient factors	Misinterpreted/misread equipment	
**Violations**	Distracting behavior	Violation of policy/procedures/standard of care	
**Preconditions for Unsafe Acts**			
**Technological environment**	Inadequate/unclear/outdated policies/procedures/checklists	Inadequate/defective warnings/alarms	Failures of information technology
**Adverse mental states**	Task overload	Mental fatigue	Perceived haste/pressure to complete task
**Adverse psychological states**	Inadequate rest/sleep	Self-medicating	Medical illness
**Physical/Mental limitations**	Lack of aptitude to operate task	Limited experience/proficiency	Lack of technical procedural knowledge
**Crew resource management**	Inaccurate information provided	Failure to warn/disclose critical information	Verification techniques not used
**Unsafe Supervision**			
**Inadequate supervision**	Inadequate oversight	Inadequate mentoring/coaching	Inadequate training
**Planned inappropriate operations**	Failure to match staff competency with the task	Poor crew pairing	
**Failed to correct a problem**	Failed to initiate corrective action	Failed to review and revise a policy/procedure	Failed to ensure problem was corrected
**Supervisory violations**	Authorized hazardous operation	Failed to enforce policies/procedures	
**Organizational Influence**			
**Resource management**	Inadequate staffing	Budgetary constraints	Failure to correct known design flaws
**Organizational climate**	Organizational values, beliefs, attitudes	Organizational customs	Norms and rules
**Organizational process**	Established safety programs/risk management programs	Management’s monitoring and checking of resources, climate, and processes to ensure a safe work environment	Operational tempo

## Data Availability

The data presented in this study are available on request from the corresponding author. The data are not publicly available due to privacy reasons.
